# Age-Specific Differences in Association Between Personality and Changes in Outing Behaviors During the COVID-19 Pandemic in Japan: Cross-Sectional Web-Based Questionnaire Survey

**DOI:** 10.2196/63120

**Published:** 2025-06-12

**Authors:** Kaori Yamaguchi, Takemi Akahane, Emi Yasuda, Manabu Akahane

**Affiliations:** 1 Department of Health and Welfare Services, National Institute of Public Health, 2-3-6, Minami, Wako City, 3510197, Japan, 81 484586159; 2 Department of Gastroenterology, Nara Medical University, Kashihara, Japan

**Keywords:** lockdown, personality, outing behaviors, internet survey, public health, Japan

## Abstract

**Background:**

The outbreak of COVID-19 in 2019 led governments worldwide to introduce various public health measures, which included restrictions on travel and public gatherings, effectively reducing the spread of the virus and associated mortality rates. In Japan, nonlegally binding restrictions on outings effectively curbed infections, as in other countries. However, the restrictions impacted lifestyles, including reduced physical activity, increased frailty, and overeating issues, beyond the effect of preventing the spread of infection. Various factors such as personality, age, and cultural norms influenced outing behavior during the pandemic, which varied by activity type.

**Objective:**

To elucidate the association between personality traits and changes in outing behaviors during the COVID-19 pandemic, as well as to clarify age-specific differences in outing behaviors, focusing on different types of outings.

**Methods:**

A cross-sectional survey was conducted using a web-based questionnaire in January 2021, when Japan announced its second emergency declaration during the pandemic. Overall, 1236 participants were recruited, with an equal number of participants for each gender and 10-year age group. The survey included questions regarding changes in the frequency of three types of outings—medical institution visits, eating out, and traveling—in addition to participants’ personality traits, such as sociability and morality. Multinomial logistic regression analysis was performed to analyze the association between personality traits and changes in different outing behaviors. Stratified analysis by age group was also performed.

**Results:**

The findings revealed that 790 participants reported no change in medical institution visits, although the frequency of eating out and traveling decreased during the pandemic. Regarding an age-wise comparison, a higher percentage of older people reported no change in medical institution visits but reported a decrease in eating out and traveling than younger people. Multinomial logistic regression analysis stratified by age showed that sociable people were more likely to report a decrease in the frequency of medical institution visits and an increase in the frequency of eating out (odds ratio [OR] 1.92, 95% CI 1.36‐2.71, *P*<.001; OR 2.57, 95% CI 1.19‐5.54, *P=*.016, respectively), and participants with a strong sense of responsibility were more likely to report a decrease in the frequency of traveling (OR 1.76, 95% CI 1.14‐2.72, *P=*.011) among younger adults. Among older adults, strongly responsible individuals were less likely to eating out frequently (OR 2.56, 95% CI 1.12‐5.82, *P=*.026).

**Conclusions:**

We examined various behavioral changes observed during the pandemic for different types of outings and their associations with personality traits, as well as differences between age groups. The findings could help promote an understanding of how to effectively communicate and engage in appropriate behaviors in public health emergency settings.

## Introduction

Various social measures were implemented to curb the global spread of COVID-19, including maintaining physical distance, hand hygiene, wearing masks, and restrictions on domestic and international travel, with evidence of their effectiveness has been documented [1-3]. For example, Lau et al [4] reported that the doubling time of infections increased from 2 to 4 days after a lockdown was imposed in Wuhan, China. Fowler et al [5] reported that in the United States, a stay-at-home order was associated with a more than 30% reduction in weekly COVID-19 cases after 1 week and an approximately 60% reduction in weekly fatalities after 3 weeks in localities that implemented the measure.

Although travel and public gathering restrictions effectively controlled the spread of infection, they led to some unintended effects on the lifestyles of adults, especially older adults, resulting in health impairments [6-10]. For instance, Shinohara conducted a prospective six-month cohort study immediately after the first travel and public gathering restrictions in Japan, reporting an increase in frailty among older people [6]. Esquinas reported a decrease in physical activity and an increase in sedentary lifestyle for community-dwelling older people several weeks after a lockdown was implemented in Spain [7]. In a survey conducted after the implementation of restrictions accompanying the pandemic in Slovakia, Lorková et al [8] reported changes in eating habits, demonstrating that more than 30% of Slovak adults, both men and women, indulged in overeating, and more than half reported weight gain. Tavolacci et al [9] reported that the proportion of students engaging in physical activities decreased, and the risk of depression increased for men among students of a French university. Moreover, unemployment and reduced income during the lockdown affected the economic and psychological well-being of young adults in 6 countries across different regions [10].

Restrictions on outings also impacted disease prevention efforts, with the number of people receiving medical screenings decreasing during the pandemic [11-13]. Bakouny et al [11] conducted a hospital survey in Massachusetts during the pandemic and found that the number of patients receiving cancer screening and diagnosis had decreased in the previous 3 months compared with the 3 months before the pandemic. A trend analysis in Spain also revealed a decline in the number of cancer diagnoses during and after the lockdown among youth, adults, and older adults at primary care clinics [12]. Furthermore, a study in Japan found a decrease in the number of patients receiving cancer screening under the restrictions and its association with anxiety triggered by COVID-19 [13]. Such trends are concerning because delays in disease detection and treatment could lead to increased mortality rates in the long term [14].

Restrictions on outings in Japan were aimed at preventing the spread of infection by reducing person-to-person contact and, consequently, large-scale cluster infections at specific locations or events with large groups of people, which would have further disrupted the medical system [15,16]. The restrictions were not legally binding but rather represented a declaration of emergency urging citizens to change their outing behaviors to prevent the spread of infections. This was implemented multiple times (each time lasting from 1 to 3 weeks) between 2020 and 2021 in all prefectures across the country, which effectively helped curtail infections [17]. Although the declaration was not legally binding, Japanese people strictly adhered to the restrictions on going out, and the streets were largely deserted.

Given the wide impact of behavioral restrictions beyond preventing the spread of infection, their appropriate implementation is important. For implementation to be adequate, it must be recognized that people’s preventive behavior is influenced by factors such as their health and information literacy, gender, age, and personality traits [18-30].

Personality traits have been reported to be associated with various types of behaviors related to the pandemic [25-28,30]. Previous studies have highlighted the association between personality traits, such as morality, conscientiousness, and neuroticism, and COVID-19 prevention behavior, such as maintaining hygiene and social distancing [25-27]. There have also been reports on the association of outing behaviors with personality traits such as modesty, honesty, sociability, and extraversion [23,28,29]. Additionally, studies have highlighted the relationship between preventive behavior and age [20-22,30]. Older adults positively adopted preventive measures and had higher health-related literacy [20,21]. Furthermore, among older people, unlike younger people, perceptions of the seriousness of the situation were associated with preventive behavior [22].

Preventive behavior is also influenced by culture, as Abuliezi et al [31] reported, Japanese medical students performed significantly better during prevention measures than their American counterparts. Previous studies have reported that the Japanese are sensitive to social pressure, have a strong tendency to avoid negative evaluations from others, and adhere to infection-prevention protocols, driven by social norms and cultural background [31-34].

Furthermore, regarding outing behaviors during the pandemic, different behavioral changes were reported depending on the type of outing. For instance, eating out and leisure activities decreased, whereas visits to neighborhood parks increased [23,24,35,36]. These findings indicate that the impact of the pandemic on outings may have varied depending on the type of activity.

Preventive behavior, including restrictions on outings, is influenced by various factors such as personality, age, and culture, with behavior also differing depending on the type of outing; however, no findings considering them collectively have been reported. Therefore, we aimed to clarify the association between personality traits and changes in outing behaviors during the COVID-19 pandemic, as well as determine age-specific differences, focusing on three types of outings: medical institution visits, eating out, and traveling. The findings could serve as useful tools when considering effective implementation that takes into account population characteristics for preparedness for emergencies such as infectious disease epidemics.

## Methods

### Study Design, Setting, and Participants

A cross-sectional analysis was conducted using a web-based questionnaire survey on January 27‐29, 2021, when the COVID-19 pandemic was ongoing in Japan. The central government had declared a state of emergency during the survey period in 11 of the 47 prefectures (Tochigi, Saitama, Chiba, Tokyo, Kanagawa, Gifu, Aichi, Kyoto, Osaka, Hyogo, and Fukuoka) according to the number of infections, situation of the medical system, and the level of development of monitoring systems [15-17]. It was the second declaration, 8 months after the first emergency declaration in Japan [15-17].

An internet-based research company, Macromill, Inc, Japan was engaged to recruit participants and collect responses and the company’s registered monitors include over 30 million individuals living in Japan. The survey included 1236 participants aged between 20 and 79 years from across Japan. The company was responsible for recruiting participants and collecting responses. The participants were randomly selected, and an email was sent inviting them to take the survey. An equal number of participants from both genders and 10-year age groups were recruited, with registrations closing once the targeted sample size was achieved. The participants completed the questionnaires through email and received a small reward, as determined by the company.

### Data Collection

Cross-sectional data from the web-based questionnaire included information on participants’ backgrounds, health-related items, changes in outing behaviors due to the pandemic, and personality traits (Table 1). Economic status was reported to influence outing behavior during the pandemic [37], and the analysis included participants’ income as an adjustment variable. Changes in the frequency of three types of outings were included: medical institution visits, eating out, and traveling. Scoring was performed on a five-point Likert scale (1=Increased, 2=Somewhat increased, 3=No change, 4=Somewhat decreased, 5=Decreased).

**Table 1.** Items included in the questionnaire

Question^a ^	Response
What is the number of people, including yourself, living in your house?	1: 1, 2: 2, 3: 3, 4: 4, 5: 5, 6: 6 or more
Please indicate the family members living with you. (Multiple answers allowed).	1: Infants or toddlers (not attending nursery or kindergarten)2: Infants or toddlers (attending nursery or kindergarten)3: Students (elementary school–junior high school)4: Students (senior high school–college, including vocational school) (attending school in the same prefecture)5: Students (senior high school–college, including vocational school) (attending school in another prefecture)6: Adults7: Older adults (youngest-old: aged 65‐74)8: Older adults (middle-old: above 75)9: Other (please specify)
Please tell us about your chronic disease(s). Choose from options 1‐6 above for all except “Other.”	
Hypertension	1: Undergoing treatment (oral medication), 2:Follow-up (regular hospital visits only), 3: Follow-up (without hospital visits), 4: Untreated, 5: Treated, 6: Not applicable.
Diabetes mellitus	Same as above
Heart disease	Same as above
Cerebrovascular disease	Same as above
Malignant tumor (cancer, leukemia, etc)	Same as above
Respiratory disease (asthma, emphysema, etc)	Same as above
Dyslipidemia (hyperlipidemia)	Same as above
Liver disease	Same as above
Dementia	Same as above
Mental illness (depression, etc)	Same as above
Other (please specify)	Same as above
Please tell us about your current condition.	1: I am physically healthy, 2: I am mentally healthy, 3: I am financially stable.
Please tell us about yourself.	
I am diligent.	1: Strongly disagree, 2: Disagree, 3: Somewhat disagree4: Somewhat agree, 5: Agree, 6: Strongly agree
I am sociable.	Same as above
I have a strong sense of responsibility.	Same as above
I have a strong sense of morality.	Same as above
I am cooperative.	Same as above
I am honest.	Same as above
Please tell us about the changes you experienced in the following compared with last year.	
Medical institution visit frequency	1: Increased, 2: Somewhat increased, 3: No change,4: Somewhat decreased, 5: Decreased
Eating out frequency	Same as above
Traveling frequency	Same as above

a age, gender, marital status, employment status, yearly household income, and prefectures were prerecorded during the members’ registration with the survey panel.

The survey referred to the Japanese version of the Personality Inventory, which is based on the “Big Five” model to assess personality traits [38]. Japanese personality traits “sense of morality,” “diligence,” and “strong sense of responsibility” were also included because these could influence preventive behaviors toward COVID-19 [26,39]. To minimize the burden on participants, the items were grouped into six categories: “diligent,” “sociable,” “cooperative,” “honest,” “strong sense of responsibility,” and “strong sense of morality.” Regarding the response, the original seven-point Likert scale was modified to a six-point scale for aggregation and analysis purposes [15,40]. The participants were asked to indicate their agreement levels for each personality trait using the scale (1=Strongly disagree, 2=Disagree, 3=Somewhat disagree, 4=Somewhat agree, 5=Agree, 6=Strongly agree).

### Data Analysis

A multinomial logistic regression was performed to analyze the association between personality traits and changes in outing behaviors. After verifying multicollinearity, all independent variables representing personality traits were simultaneously inputted. Additionally, a stratified analysis was performed by age group. We classified participants into two groups: younger and older adults. Considering the number of participants in each age group, the older group included individuals aged 60 and above.

Regarding the variables, the scoring for each outing was classified into three groups—increase (1=Increased and 2=Somewhat increased), no change (3=No change), and decrease (4=Somewhat decreased and 5=Decreased)—which were used as dependent variables, and “no change” was used as the baseline category for multinomial logistic regression. The responses for personality traits were categorized into two groups—applicable (4=Somewhat agree, 5=Agree, and 6=Strongly agree) or not applicable (1=Strongly disagree, 2=Disagree, and 3=Somewhat disagree)—which were considered as independent variables. The following variables were included as covariates: gender, age, marital status, living alone, living with children (aged younger than 15 years), living with older adults (aged older than 65 years), low household income (less than JPY 4 million (USD 36500) annually), self-reported disease, self-reported health, employment status, and emergency declaration status.

For all statistical analyses, we used STATA 17/SE (StataCorp LLC) with a statistical significance of *P*<.05.

### Ethical Considerations

This study was approved by the Ethics Committee of the National Institute of Public Health, Japan (NIPH-IBRA#12302, approval date: November 17, 2020), in accordance with the Declaration of Helsinki. All participants provided informed consent for data collection and storage. Written informed consent for study participation was obtained upon registration. The web-based questionnaire survey was conducted by an authorized survey company in adherence with personal information protection regulations. Anonymized data were obtained from the company after survey completion.

## Results

Table 2 presents the participants’ background characteristics and changes in outing behavior before and during COVID-19. The mean (SD) age of the 1236 participants was 49.4 (16.5) years; 618 (46.6%) were men. In terms of personality traits, over 75% of the participants exhibited attributes such as being “honest” (n=1028, 83.2%), having a “strong sense of responsibility” (n=957, 77.4%), and having a “strong sense of morality” (n=965, 78.1%).

**Table 2.** Characteristics of participants and changes in outing behaviors.

Characteristics	All participantsn=1326	Younger adultsn=824	Older adults (older than 60 years)n=412
Sociodemographic factors			
Age (years), mean (SD)	49.4 (16.5)	39.9 (11.1)	68.3 (5.2)
Gender (men), n (%)	618 (46.6)	412 (50.0)	206 (50.0)
Annual household income (<JPY 4 million (USD 36500)), n (%)	364 (29.5)	198 (24.0)	166 (40.3)
(unknown), n (%)	275 (22.3)	217 (26.3)	58 (14.1)
Employment (current), n (%)	749 (60.6)	602 (73.1)	147 (35.7)
Marital status (married), n (%)	446 (36.1)	367 (44.5)	79 (19.2)
Living alone, n (%)	207 (16.8)	149 (18.1)	58 (14.1)
Living with children aged 15 years and younger, n (%)	267 (21.6)	256 (31.1)	11 (2.7)
Living with older adults aged 65 years and older, n (%)	392 (31.7)	148 (18.0)	244 (59.2)
Self-reported disease (more than one disease in treatment), n (%)	542 (43.9)	268 (32.5)	274 (66.5)
Self-reported health (good), n (%)	928 (75.1)	606 (73.5)	322 (78.2)
Emergency declaration^a^ when conducting the survey (in progress), n (%)	783 (63.4)	506 (61.4)	277 (67.2)
Personality traits			
Diligent (applicable), n(%)	709 (57.4)	412 (50.0)	297 (72.1)
Sociable (applicable), n(%)	554 (44.8)	357 (43.3)	197 (47.8)
Having a strong sense of responsibility (applicable), n(%)	957 (77.4)	594 (72.1)	363 (88.1)
Having a strong sense of morality (applicable), n(%)	965 (78.1)	596 (72.3)	369 (89.6)
Cooperative (applicable), n(%)	887 (71.8)	547 (66.4)	340 (82.5)
Honest (applicable), n (%)	1028 (83.2)	644 (78.2)	384 (93.2)
Frequency of outing behaviors			
Medical institution visits, n (%)			
Increased	88 (7.1)	68 (8.3)	20 (4.9)
Remained unchanged	790 (63.9)	496 (60.2)	294 (71.4)
Decreased	358 (29.0)	260 (31.6)	98 (23.8)
Eating out, n (%)			
Increased	52 (4.2)	45 (5.5)	7 (1.7)
Remained unchanged	311 (25.2)	217 (26.3)	94 (22.8)
Decreased	873 (70.6)	562 (68.2)	311 (75.5)
Traveling, n (%)			
Increased	28 (2.3)	25 (3.0)	3 (0.7)
Remained unchanged	273 (22.1)	201 (24.4)	72 (17.5)
Decreased	935 (75.7)	598 (72.6)	337 (81.8)

a Following the outbreak of COVID-19, a state of emergency was declared in Japan during the survey period in 11 of the 46 prefectures: Tochigi, Saitama, Chiba, Tokyo, Kanagawa, Gifu, Aichi, Kyoto, Osaka, Hyogo, and Fukuoka.

Regarding changes in outing behavior, participants’ responses indicated that the frequency of medical institution visits remained unchanged (n=790, 63.9%). However, the majority of the participants reported a decrease in the frequency of eating out (n=873, 70.6%) and traveling (n=935, 75.6%).

Comparing younger and older age groups, a higher proportion of older individuals reported no change in medical institution visits, while a higher proportion of younger individuals reported increased or decreased medical institution visits compared with the older adult group. Regarding eating out and traveling, a lower proportion of younger individuals reported a decrease in eating out and traveling, while a higher percentage reported an increase in eating out and traveling, compared with the older group.

Tables3  4 present the odds ratios (OR) and 95% Confidence Interval (CI) determined through multinomial logistic regression analysis and the stratified analysis for younger and older adults. The analysis examined the association between six personality traits and changes in the frequency of outing behaviors, such as increase, no change, and decrease, in three different types of outings and those for younger and older age groups. Participants with each of these traits were compared with those without these personality traits.

**Table 3.** Association between personality traits and changes in outing behaviors (among all participants, n=1326).

Variable	Odds ratio (95% CI)^a^	*P* value
Medical institution visits		
Diligent		
Increase	0.71 (0.41-1.23)	.223
Decrease	0.93 (0.69-0.69)	.623
Sociable		
Increase	0.86 (0.51-1.46)	.582
Decrease	1.55 (1.17-2.06)	.002
Responsibility		
Increase	1.56 (0.86-2.83)	.144
Decrease	1.17 (0.77-1.77)	.473
Morality		
Increase	0.98 (0.54-1.78)	.960
Decrease	1.39 (0.93-2.08)	.112
Cooperative		
Increase	1.34 (0.73-2.49)	.346
Decrease	0.91 (0.65-1.26)	.561
Honest		
Increase	0.60 (0.33-1.10)	.098
Decrease	1.08 (0.71-0.71)	.714
Eating out		
Diligent		
Increase	1.13 (0.51-2.52)	.763
Decrease	1.05 (0.77-1.42)	.774
Sociable		
Increase	2.04 (1.02-4.08)	.045
Decrease	1.09 (0.81-1.47)	.570
Responsibility		
Increase	1.25 (0.50-3.11)	.630
Decrease	1.33 (0.90-1.94)	.149
Morality		
Increase	0.71 (0.34-1.49)	.367
Decrease	0.98 (0.66-1.45)	.922
Cooperative		
Increase	1.07 (0.52-2.21)	.857
Decrease	1.09 (0.78-1.53)	.600
Honest		
Increase	0.94 (0.41-2.14)	.881
Decrease	1.33 (0.91-1.97)	.144
Traveling		
Diligent		
Increase	1.83 (0.65-5.15)	.249
Decrease	1.01 (0.73-1.40)	.936
Sociable		
Increase	1.39 (0.50-3.84)	.523
Decrease	1.31 (0.96-1.80)	.092
Responsibility		
Increase	1.46 (0.54-3.97)	.456
Decrease	1.81 (1.22-2.68)	.003
Morality		
Increase	0.59 (0.22-1.59)	.298
Decrease	1.41 (0.95-2.08)	.086
Cooperative		
Increase	1.02 (0.39-2.65)	.974
Decrease	1.08 (0.77-1.51)	.656
Honest		
Increase	0.62 (0.24-1.59)	.318
Decrease	1.12 (0.74-1.69)	.587

a The odds ratio of decrease in outing frequency was estimated by multinomial logistic regression adjusted for age, gender, household income, employment, marital status, living alone, living with children, living with older adults, self-reported diseases in treatment, self-reported health, and emergency declaration status for all participants and each stratified group at “no change” as baseline outcome. Participants without personality traits were set as the reference for each odds ratio. CI: Confidence Interval

**Table 4.** Association between personality traits and changes in outing behaviors (stratified by age).

Variable	Younger adults (n=824)Odds ratio (95% CI)^a^	*P* value	Older adults (>60 years; n=412)Odds ratio (95% CI)^a^	*P* value
Medical institution visits				
Diligent				
Increase	0.76 (0.40-1.44)	.395	0.53 (0.20-1.40)	.198
Decrease	0.98 (0.69-1.39)	.903	0.90 (0.50-1.61)	.714
Sociable				
Increase	1.08 (0.58-2.00)	.818	0.64 (0.23-1.83)	.409
Decrease	1.92 (1.36-2.71)	<.001	1.19 (0.72-1.96)	.509
Responsibility				
Increase	1.29 (0.68-2.47)	.436	4.82 (0.47-49.84)	.187
Decrease	1.07 (0.67-1.72)	.772	1.58 (0.58-4.32)	.371
Morality				
Increase	0.87 (0.44-1.72)	.691	0.98 (0.22-4.30)	.974
Decrease	1.19 (0.76-1.87)	.445	2.38 (0.73-7.78)	.152
Cooperative				
Increase	1.27 (0.63-2.57)	.510	1.61 (0.45-5.77)	.461
Decrease	0.91 (0.63-1.33)	.639	0.89 (0.42-1.88)	.760
Honest				
Increase	0.63 (0.33-1.18)	.148	0.52 (0.09-3.06)	.469
Decrease	1.17 (0.75-1.84)	.482	0.63 (0.20-2.01)	.432
Eating out				
Diligent	** **	** **	** **	** **
Increase	1.53 (0.67-3.49)	.310	0.24 (0.02-2.73)	.250
Decrease	1.14 (0.79-1.64)	.486	0.81 (0.43-1.55)	.532
Sociable				
Increase	2.57 (1.19-5.54)	.016	1.61 (0.20-13.22)	.655
Decrease	1.13 (0.78-1.63)	.529	1.07 (0.63-1.81)	.811
Responsibility				
Increase	1.13 (0.43-2.96)	.801	―^b^	―
Decrease	1.11 (0.72-1.71)	.627	2.56 (0.12-5.82)	.026
Morality				
Increase	0.57 (0.25-1.30)	.183	―	―
Decrease	1.12 (0.72-1.74)	.619	0.60 (0.22-1.58)	.297
Cooperative				
Increase	0.98 (0.44-2.20	.968	2.35 (0.16-34.42)	.534
Decrease	0.98 (0.66-1.45)	.931	1.53 (0.80-2.93)	.195
Honest				
Increase	1.16 (0.48-2.84)	.738	0.25 (0.01-4.50)	.346
Decrease	1.49 (0.97-2.29)	.066	0.56 (0.18-1.75)	.322
Traveling				
Diligent	** **	** **	** **	** **
Increase	2.81 (0.94-8.35)	.063	―	―
Decrease	1.01 (0.69-1.48)	.956	―	―
Sociable				
Increase	1.47 (0.49-4.40)	.486	―	―
Decrease	1.38 (0.95-2.02)	.095	―	―
Responsibility				
Increase	1.14 (0.40-3.29)	.803	―	―
Decrease	1.76 (1.14-2.72)	.011	―	―
Morality				
Increase	0.43 (0.14-1.32)	.139	―	―
Decrease	1.48 (0.95-2.28)	.081	―	―
Cooperative				
Increase	1.11 (0.38-3.18)	.852	―	―
Decrease	1.03 (0.70-1.52)	.874	―	―
Honest				
Increase	0.84 (0.32-2.22)	.721	―	―
Decrease	1.10 (0.70-1.72)	.672	―	―

a The odds ratio of decrease in outing frequency was estimated by multinomial logistic regression adjusted for age, gender, household income, employment, marital status, living alone, living with children, living with older adults, self-reported diseases in treatment, self-reported health, and emergency declaration status for all participants and each stratified group at “no change” as baseline outcome (age was adjusted only for all participants). Participants without personality traits were set as the reference for each odds ratio. Values regarding the association between morality and eating out for older adults were not calculated, as the imbalance of the data with an extremely low sample size in a particular category resulted in inappropriate coefficients. The result of the association between personality traits and traveling for the older adult group was not calculated because some categories had extremely low sample sizes, which led to data imbalance, and the model did not converge appropriately. CI: Confidence Interval

b not available

In terms of the association between personality traits and outing behaviors, sociable individuals were more likely to report a decrease in the frequency of medical institution visits compared with individuals who were not sociable, for all participants and younger adults (OR 1.55, 95% CI 1.17‐2.06, *P*=.002 and OR 1.92, 95% CI 1.36‐2.71, *P*<.001, respectively). Regarding eating out, sociable individuals were more likely to report an increase among all participants and younger adults (OR 2.04, 95%CI 1.02‐4.08, *P*=.045 and OR 2.57, 95% CI 1.19‐5.54, *P=*.016, respectively), whereas participants with strong responsibility were more likely to report a decrease among the older adults group (OR 2.56, 95% CI 1.12‐5.82, *P*=.026). Regarding traveling, participants with strong responsibility were more likely to report a decrease among all participants and younger adults (OR 1.81, 95% CI 1.22‐2.68, *P=*.003 OR 1.76, 95% CI 1.14‐2.72, *P=*.001, respectively).

Values regarding the association between morality and eating out for older adults were not calculated, as the imbalance of the data with an extremely low sample size in a particular category resulted in inappropriate coefficients. The result of the association between personality traits and traveling for the older adult group was not calculated because some categories had extremely low sample sizes, which led to data imbalance, and the model did not converge appropriately.

## Discussion

### Principal Findings

The analysis revealed two main findings. First, the impact of COVID-19 on outing behavior varied depending on the type of activity. Although most participants reported no change in the frequency of medical institution visits, the majority reported a decrease in the frequency of eating out and traveling. To compare the age groups, a higher percentage of older adults reported no change in medical institution visits and a decrease in eating out and traveling compared with younger adults. Second, the impact of COVID-19 on outing behavior was associated with particular personalities. Participants with a sociable personality were more likely to report a decrease in the frequency of medical institution visits and an increase in the frequency of eating out, while participants with a strong sense of responsibility were more likely to report a decrease in the frequency of traveling. The association between changes in outing behavior and personality differed by age group. Younger adults showed similar trends across all participants; meanwhile, older adults with a strong sense of responsibility were more likely to report a decrease in the frequency of eating out.

For visits to medical institutions, previous studies have revealed that seeking emergency treatments remained unchanged and screening examinations decreased during the pandemic [11,12,36,41]. Examining visits to medical institutions, which may include outpatient treatment, we found that this aspect remained unchanged for most participants during the pandemic. Although telemedicine was introduced in Japan in 2018, its application remained limited even during the pandemic; thus, its impact on medical visits was considered minimal, if any. Regarding eating out, studies in Japan [24], Poland [38], and the United States [23] found a reduction in frequency during lockdowns, consistent with our findings. Similarly, for travel, previous studies reported that the COVID-19 pandemic and infection control measures led to a decrease in long-distance travel [42-45], consistent with our findings. Regarding the variation in results by age group, Harrera et al surveyed during the pandemic in Spain and clarified the association between older age and higher frequency of preventive practices, higher health literacy, and greater concern about COVID-19 [22]. They also reported that increased adherence to preventive practices was associated with being female, having greater concern about COVID-19, and seeking more information among the younger generation; meanwhile, for the older generation, it was associated with greater concern about COVID-19 [22]. Concerns about COVID-19 might also have influenced our result that a higher proportion of older adults reduced the frequency of eating out and traveling.

Importantly, three types of outing behaviors were simultaneously investigated, revealing that medical institution visits remained unchanged, whereas eating out and traveling decreased, with the trend differing among age groups. This suggests that the impact of the pandemic on outing behavior varied depending on necessity.

The findings also indicated that outing behaviors in response to COVID-19 were associated with particular personality traits. Personality traits have been reported to be associated with various behaviors related to the COVID-19 pandemic [25,26,29,30]. For example, Qian and Yahara [26] determined an association between preventive behaviors and personality traits such as extraversion, neuroticism, openness, conscientiousness, and agreeableness, as well as a sense of morality. Hygiene and social distancing behaviors have also been associated with personality traits such as extroversion, honesty, modesty, conscientiousness, and neuroticism [25,30]. In a study conducted in Italy, Costantini et al [30] identified personality traits that predicted compliance with COVID-19 prevention measures, including outing behavior. Their findings revealed that the frequency of outing behavior during the pandemic was negatively associated with modesty and creativity and positively associated with liveliness and sociability. The findings on eating out among younger adults align with those of this study. Although the previous study found a positive association between the frequency of outings and sociable personalities, we revealed that sociable people were more likely to report a decrease in the frequency of one type of outing: medical institution visits. This inconsistency may attributed to the evaluations in previous studies focusing on “unnecessary outings” [30].

Cultural aspects were reported to influence behavior during the pandemic. Abuliezi et al [31] reported the characteristics of preventive behavior among Japanese students compared with those in the United States. They determined that Japanese medical students performed significantly better in preventive behavior, although perceived health competence was lower, which means people do not have the confidence to manage their health through health-related habits [31]]. Furthermore, Japanese people tend to avoid social pressure and negative evaluations from others [33,34]. To sum up these cultural aspects, the finding regarding the association between sociable personality and an increase in the frequency of eating out among younger adults may be attributed to peer pressure to eating out or such a trend among peers.

Furthermore, changes in people’s perceptions and behaviors after the pandemic have been reported [46]. Although a better attitude and higher knowledge levels were observed, adherence to protocols remained inconsistent one year after the outbreak of the pandemic among younger adults aged 16‐30 years in Bangladesh [46]. This survey was also conducted a year after the first emergency declaration; therefore, the results might be influenced by changes in people’s perceptions and attitudes toward the pandemic over time. Considering the results and the survey periods of this study, concerns regarding COVID-19 among older adults might have changed their outing behavior even during the early stages of the pandemic. For a deeper understanding, further analysis would be required to clarify how the experience of the pandemic and repeated emergency declarations affected attitudes and behaviors.

### Limitations

Although this study effectively elucidates the association between personality traits and different types of outings during COVID-19 and differences by age groups, there are certain limitations that are worth mentioning. First, the participants responded to the questions based on their memories of the situation prior to the pandemic, and the actual number of outings before and during the pandemic was not observed; thus, response bias is possible. Furthermore, response bias resulting from the high social desirability of Japanese people could have affected the results [47]. Second, given the equal recruitment across age groups and genders, the participants did not represent the general population structure of Japan. In accordance, it should be noted that descriptive statistics regarding changes in the frequency of outing behavior do not represent the situation in Japan. Finally, we cannot eliminate the possibility of selection bias, as we only included participants registered with an internet panel company who had access to the internet and could respond on the web. Nevertheless, panel-based surveys have been widely used in recent years [48-50]. In addition, the participants received a small cash reward for completing the survey, which could have affected the randomness of the sample. However, because the participants were randomly selected from a large number of people registered with the engaged company across Japan, bias is expected to be minimized.

### Conclusions

We examined various behavioral changes observed during the pandemic for different types of outings and their associations with personality traits and differences between age groups. The findings can help in understanding how to effectively communicate and promote appropriate behaviors in public health emergency settings.

## Abbreviations

OR: odds ratio
CI: Confidence Interval
